# Sharper, straighter, stiffer, stronger: sexually dimorphic bill shape enhances male stabbing performance in the green hermit hummingbird (*Phaethornis guy*)

**DOI:** 10.1242/jeb.250769

**Published:** 2025-11-10

**Authors:** Felipe Garzón-Agudelo, Lucas Mansfield, Kevin Epperly, Alejandro Rico-Guevara

**Affiliations:** ^1^ Centro de Investigación Colibrí Gorriazul, Fusagasugá, Cundinamarca 252217, Colombia; ^2^ Department of Biology, University of Washington, Seattle, WA 98195, USA; ^3^ Burke Museum of Natural History and Culture, University of Washington, Seattle, WA 98105, USA

**Keywords:** Beak sexual dimorphism, Fighting performance, Bird weapons, Lekking hummingbirds, 3D geometric morphometrics, Finite element analysis

## Abstract

Bill sexual dimorphism has been primarily linked to differential use of food resources between sexes. However, intrasexual selection has been suggested to also influence hummingbird bill morphology. Males of *Phaethornis longirostris* (long-billed hermit) possess sharp, elongated, dagger-like bill-tips that enhance puncturing ability and territory defense during male–male lekking combat. Yet, the prevalence of weaponized bills in hermits and the impact of bill shape on fighting performance remain unexplored. We employed 3D modelling and finite element analysis to explore bill dimorphism and stabbing performance in another lekking hummingbird: *Phaethornis guy* (green hermit)*.* Our results reveal that *P. guy* exhibits a male-specific bill-tip dagger, and that males’ straighter bills show greater biomechanical performance during stabbing tests, transmitting forces more efficiently (strain energy) and reducing the risk of breakage (von Mises stress). These findings provide further evidence of bill-tip weaponry and support a role of agonistic interactions in the evolution of bill dimorphism.

## Teaser

**Highlighted Article:** Male green hermit hummingbirds possess sharper, straighter and stronger bills than their female counterparts, providing evidence of the role of agonistic interactions in the evolution of bill dimorphism.

## INTRODUCTION

Sexually dimorphic traits, beyond those directly related to sexual reproduction (e.g. reproductive organs), are widespread across animals and are linked to species' life histories (Janicke and Fromonteil, 2021; Rico-Guevara and Hurme, 2019). These intraspecific differences manifest in traits such as coloration, body size, or ornamentation, usually linked to sexual selection (Andersson, 1994; Emlen, 2008). Additionally, ecological mechanisms, including resource competition (Butler et al., 2000) and differential responses to environmental gradients (Hendry et al., 2006), have also been shown to influence phenotypic differences between sexes. For bird bills specifically, intrasexual selection, a process wherein individuals compete with same-sex conspecifics for reproductive opportunities, has also been linked to the evolution of secondary sexual dimorphism (Rico-Guevara and Hurme, 2019). Studying the details of sexual dimorphism within a species is essential for understanding its potential evolutionary mechanisms.

Hummingbirds make excellent subjects for studying sexual dimorphism and its causal mechanisms, as they often exhibit pronounced sexual differences (e.g. in plumage: Bleiweiss, 1997; Beltrán et al., 2022). Bill sexual dimorphism is common across hummingbirds (Bleiweiss, 1999; Temeles et al., 2010; Berns and Adams, 2013), with females typically having longer bills than males, except in many species of the hermit clade (Phaethornithinae), wherein males tend to be the longer-billed sex (Bleiweiss, 1999; Temeles et al., 2010). Hermits also frequently exhibit strong sexual dimorphism in bill curvature, particularly in larger species, wherein females bear curvier bills than their male counterparts (Temeles et al., 2010). These sexual differences have been attributed to feeding (i.e. intersexual resource partitioning: Temeles et al., 2010), as variation in bill curvature and length enables hummingbirds to exploit different floral resources more efficiently (Rico-Guevara et al., 2021).

Hermit hummingbirds form leks during the breeding season, where males engage in aggressive behaviors, including physical combat (Snow, 1973; Stiles and Wolf, 1979; Harger and Lyon, 1980; MacDougall-Shackleton and Harbison, 1998). In long-billed hermits (*Phaethornis longirostris*), video-documented combat includes a hovering male charging a perched rival and stabbing it in the throat with the bill-tip, followed by a chase and the intruder's retreat (movie A2 in Rico-Guevara and Araya-Salas, 2015). Adult long-billed hermit males exhibit dagger-like bill tips, characterized by a sharp and elongated maxillary tip, which enhances puncturing ability and consequently increases success in defending lek territories (Rico-Guevara and Araya-Salas, 2015). It has been suggested that during stabbing, the straighter bills of males could transmit more force without bending, while their pointer bills would better transform that force into puncturing than blunter bills (Rico-Guevara and Araya-Salas, 2015; Rico-Guevara et al., 2019); however, no studies have tested these hypotheses. The relationship between male bill puncturing ability and lekking behavior suggest that sexual dimorphism in long-billed hermits is influenced by intrasexual selection, promoting more weaponized bills (Rico-Guevara and Araya-Salas, 2015). This idea provides a complementary hypothesis to intersexual resource partitioning (Temeles et al., 2010) and stands as a seemingly rare example of intrasexually selected weaponry in birds (Rico-Guevara and Hurme, 2019).

To further examine the weaponization hypothesis for bill sexual dimorphism, we investigated the green hermit (*Phaethornis guy*), a large species that displays lekking fighting behavior (Snow, 1973), bill sexual dimorphism (i.e. longer and straighter bills in males; Temeles et al., 2010) and is closely related to long-billed hermits (McGuire et al., 2014), known bearers of weaponized bills (Rico-Guevara and Araya-Salas, 2015). Studying the green hermit provides an independent test of whether weaponized bills are restricted to long-billed hermits or occur more broadly, potentially indicating that such morpho-functional traits are common among large, lekking hermits with sexually dimorphic bills (as both of these species are). We explored bill sexual dimorphism and stabbing performance using 3D models and finite element analysis, a method for simulating how structures respond to applied forces. We hypothesized that the straighter bills of green hermit males perform better as stabbing weapons than those of females, and expected male bills to be structurally stronger, more efficient at transmitting forces, and more resistant to buckling under the application of axial loads simulating stabbing behavior. Furthermore, we expected intersexual differences in bill-tip sharpness, indicative of male-specific daggers.

## MATERIALS AND METHODS

### Specimen information and photogrammetry modeling

We selected eight female and eight male green hermit (*Phaethornis guy* Lesson 1833) specimens, housed at the Burke Museum's ornithology collection (see specimen IDs in Table S1). All selected specimens had well-preserved, undamaged bills, and were classified as adults based on maxillary corrugations (Yanega et al., 1997), the relative gonad size (Stiles, 1980), and the absence of Bursa of Fabricius (Rhodes et al., 2022). The male specimens exhibited two plumage varieties, being either gray-chested or green-chested individuals. Prior research indicates that the gray-chested plumage is lost over the course of 4 years, indicating that our gray-chested birds are younger adult males (Snow, 1974; Stiles and Wolf, 1979). However, we did not consider male color variation in our analyses because of the limited sample size. We generated three-dimensional (3D) models of the bills using high-resolution 3D digital photogrammetry following the methods of Medina et al. (2024). Briefly, for photogrammetry data collection, we used six Sony α7R mirrorless cameras with 5320×7968 pixel resolution, each equipped with Sony 90mm macro lenses and synchronized via remote trigger (Vello, New York City, United States). For each specimen, we captured at least 480 photographs from multiple angles. To align and scale the models, we included pairs of printed coded targets with centers placed 20 mm apart (see fig. 2 in Medina et al., 2024). The photogrammetry models resulting from this process had a resolution of 0.1 mm (see fig. 3 in Medina et al., 2024). To avoid potential measurement error due to differential separation of the jaws across individuals, likely introduced as a specimen preparation artifact, we used only the maxilla in our analyses (hereafter, bill refers only to the maxillary rhamphotheca or ‘upper bill’).

### Bill sexual dimorphism

**Fig. 1.**
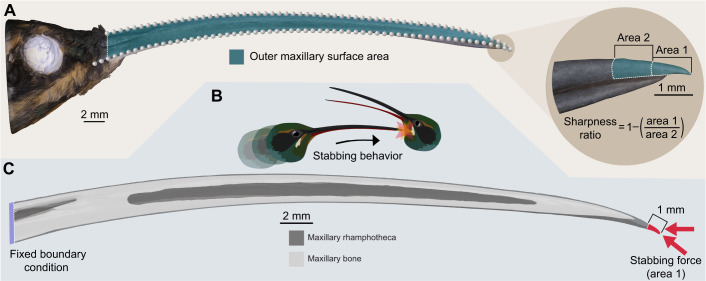
**Bill morphological characterization and stabbing behavior simulation in *Phaethornis guy*.** (A) 3D photogrammetry model (left), showing landmarks used to quantify bill shape variation (gray spheres) and selected area to quantify surface area (green). Landmarks on the culmen were used to measure bill arc length and curvature (arc:chord ratio). Close-up of the bill-tip (right) showing the outer maxillary areas used to calculate the sharpness ratio. Area 1 captures the area of the distal-most millimeter of the bill, and area 2 the second-most distal millimeter. (B) *P. guy* hypothetical stabbing behavior based on *P. longirostris* fight (movie A2 in Rico-Guevara and Araya-Salas, 2015). (C) Boundary conditions for finite element analyses simulating stabbing behavior using 3D CT models. Loads were applied at two different angles from the bill-tip.

We evaluated bill sexual dimorphism using landmark-based geometric morphometrics and by measuring bill-tip sharpness, bill curvature, arc length, and surface area (Fig. 1A). Three specimens were excluded from bill shape analysis and surface area calculations owing to peeling in the keratin of the rhamphotheca and lateral shifts in the fit of the maxilla and mandible, causing the mandible to slightly cover one of the tomia, which would affect measurements of surface area and shape analysis. Ensuring a natural alignment between upper and lower bills during specimen preparation is crucial to avoid measurement errors in morphometric analysis, particularly in small species where subtle shifts may go unnoticed.

Prior to all measurements, the 3D photogrammetry models were imported to Blender (Blender Foundation; www.blender.org), where the bill longitudinal axis was aligned parallel to the *x*-axis. We then used 3D Slicer (v.5.6.2; Fedorov et al., 2012) to digitize three contour lines on the bill models, one traced from the bill-tip along the medial-dorsal maxillary surface (in cross section, the line would go through the tallest point of the bill) to the proximal end of the exposed culmen (where the keratinous surface of the bill meets the feather line), and two other lines along the left and right tomia, from the bill-tip to the most proximal points of the tomia. Each contour curve was resampled to 60 points and intersecting points at the bill-tip were removed, giving a total of 178 points (Fig. 1A). The endpoints of the curves were treated as homologous landmarks (4 points) and the rest as semi-landmarks (174 points; Gunz and Mitteroecker, 2013). The raw landmark coordinates were imported to R for analysis. We removed non-shape variation (i.e. position, scale and orientation) through a generalized Procrustes analysis (GPA; Rohlf, 1999) using the ‘gpagen’ function in the Geomorph R package (v.4.0.10; https://cran.r-project.org/package=geomorph; Baken et al., 2021). Semi-landmarks were allowed to slide using the Procrustes distance minimization method, following the recommendations of Perez et al. (2006). As bills are bilaterally symmetric structures, the symmetric component of shape variation was extracted with the function ‘bilat.symmetry’ and used to obtain a representation of the bill shape space through a principal component analysis (PCA) with the function ‘gm.prcomp’ from Geomorph. As a result of GPA, shape (Procrustes coordinates) and size (centroid size) variables were obtained.

We used the landmark coordinates of the culmen to measure bill curvature and arc length (Fig. 1A). Using R version 4.4.1 (http://www.R-project.org/), we treated curves as two-dimensional and measured curve dimensions with the ‘coo_scalars’ function in the Momocs package (v.1.4.1; https://cran.r-project.org/package=Momocs; Bonhomme et al., 2014). Bill curvature was calculated using the arc:chord ratio (Stiles, 1995), where arc refers to the length of the upper curve of the bill from the bill-tip to the proximal point of the exposed culmen (arc length), and chord to the linear distance between these two points. Similarly to Rico-Guevara and Araya-Salas (2015), we used arc:chord ratio as it was the most conservative method for bill curvature calculation from Berns and Adams (2010).

To quantify surface area and sharpness (Fig. 1A), the models and their textures were imported into MeshLab (v.2022.02; Callieri et al., 2013). Uploading the textures allowed us to clearly visualize the tomia (cutting edges of the beak). We used the ‘subdivision surface tool’ to refine the mesh at the tomia and the bill-tip without altering the model shape, the ‘face selector tool’ to select the outer area of the maxilla from the bill-tip to the proximal end of the exposed culmen (in order to avoid differential covering of the maxilla by the face feathers at the basal end of the bill), and applied the ‘compute area of selection’ function to extract the surface area.

The dagger in the maxillary tip of the long-billed hermit is characterized by being sharper and more elongated over the mandible in adult males (Rico-Guevara and Araya-Salas, 2015). Maxillary elongation cannot be reliably measured in museum specimens, as the fit between the maxilla and mandible shifts from the natural configuration with desiccation and specimen preparation artifacts.

Several quantitative measures of morphological sharpness have been linked directly to puncture performance (Crofts et al., 2019; Anderson, 2018), including radius of curvature (Evans et al., 2005), included angle of the tip (Crofts et al., 2019), and cross-sectional area or perimeter at 1 mm from the tool tip (Schofield et al., 2016). Crofts et al. (2019) found the tip included angle to be the strongest predictor of puncture performance across multiple metrics. We evaluated sharpness using two metrics. First, we calculated a sharpness ratio based on surface area, the 3D equivalent of the ‘pointiness index’ used by Rico-Guevara and Araya-Salas (2015) (Fig. 1A). Specifically, we measured the surface area of the distal 1 mm of the bill and of the second-most distal 1 mm. The distal-most measurement was divided by the second-most distal segment and subtracted from 1 to produce the sharpness ratio, with greater values indicating sharper bills. Second, we quantified the bill-tip included angle (Fig. S1), measured in dorsal view of the bill-tip with the base of a triangle located 0.5 mm from the bill tip, with lower angles indicating sharper bills.

### Computed tomography (CT) modeling and finite element analysis

**Fig. 2.**
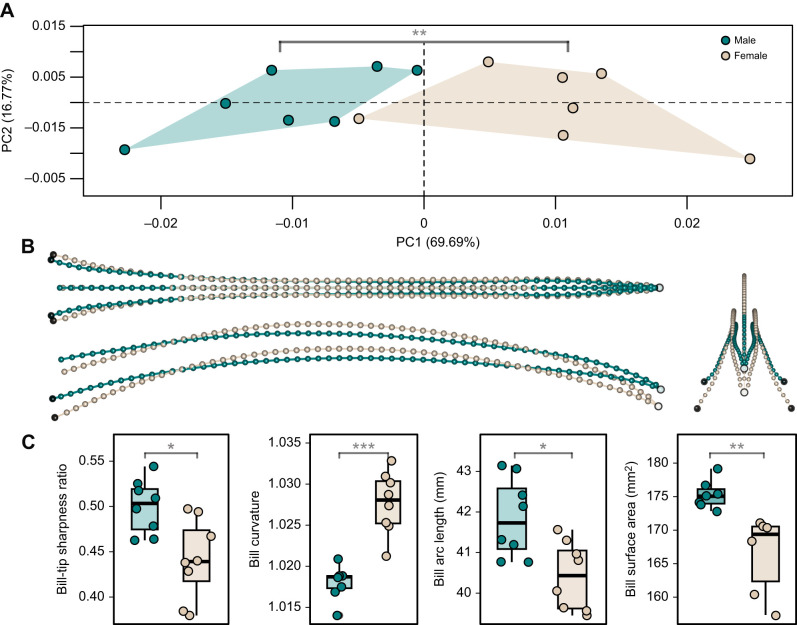
**Bills of male *Phaethornis guy* are sharper, straighter, longer and had greater surface area relative to female bills.** (A) First two principal components of the symmetric component representing bill shape space. Plot size is scaled relative to the percentage of variation explained by PCs. (B) Bill shape changes associated with the extremes of PC1 (green: male at negative end; light brown: female at positive end) in dorsal (top left), lateral (bottom left) and frontal (right) views. Shape differences are not magnified. (C) Box plots showing intersexual comparisons of bill-tip sharpness, bill curvature, arc length and surface area. Boxes show the 25–75th percentiles with median; whiskers show the 1.5× interquartile range. Level of significance (Mann–Whitney *U*): *P<0.05, **P<0.01, ***P<0.001.

To generate 3D models containing information of the inner bill structure, we CT-scanned one female and one male located at the extremes of PC1 of the bill shape space (Fig. 2A,B; Table S1). The extremes of PC1 captured the most pronounced intersexual differences in bill shape observed in our sample (see Results and Discussion). Selecting these specimens allowed us to analyze the full range of morphological variation within our sample and its potential functional implications.

Scans were performed using an NSI X5000 scanner with a voltage of 100 kV, a current of 100 µA, and a voxel resolution of 27 µm. TIFF images from CT scans were imported to 3D Slicer (Fedorov et al., 2012) and used to create 3D models of the maxillary rhamphotheca and bone in STL (stereolithography) format. Using Geomagic Studio 2014 (Geomagic Inc., Research Triangle Park, NC, USA), we isolated the bill from the head at the proximal-most point of the exposed culmen, removed any model artifacts, aligned the bill-tip and the tomia proximal ends to a common horizontal plane, and converted the STL models into computer-aided design (CAD) geometries, which are smooth surface models that facilitate the generation of high-quality finite element meshes. The resulting watertight (i.e. closed-surface) 3D models were imported into Ansys 2023 R2 (ANSYS Inc., Canonsburg, PA, USA) for linear static finite element analysis (FEA; Rayfield, 2007).

We then generated finite element meshes from the CAD models, composed of quadratic (10-noded) tetrahedral elements (female model: 878,768 nodes and 464,982 elements; male model: 923,742 nodes and 492,350 elements). Homogeneous and isotropic material properties were assigned to the meshes, with Young's moduli of 1700 MPa for the rhamphotheca and 7300 MPa for the inner bone, and a Poisson's ratio of 0.4 for both tissues, based on parameters previously used for bird bills (Soons et al., 2012a,b). Bonded contacts (*sensu* Marcé-Nogué, 2022) were used between the rhamphotheca and the bone*.* The models were constrained at the base of the bill to represent the area of attachment to the head. Axial forces were then applied to the last millimeter of the bill-tip to simulate stabbing combat behavior (Fig. 1B,C) as seen in long-billed hermits (movie 2 from Rico-Guevara and Araya-Salas, 2015). To assess performance under different conditions, forces were applied at two angles: one horizontally (horizontal stabbing) and another parallel to the axis of the bill-tip (parallel stabbing).

We conducted two types of finite element analyses: one in which we varied the magnitude of the applied forces based on empirical data (unscaled models), and another where forces were scaled according to equivalent force-to-surface-area ratios (scaled models). For unscaled analyses, we used the average forces required by the bills of long-billed hermits to pierce a skin-like material (polyvinyl chloride film) – 200 mN for females and 125 mN for adult males – as previously measured by Rico-Guevara and Araya-Salas (2015). The unscaled models allowed us to make intersexual comparisons of bill mechanical behavior while performing the hypothesized function of dagger-like bill-tips (i.e. puncturing). Scaled models, on the other hand, allow the comparison of mechanical performance due to shape alone by removing size differences (Dumont et al., 2009).

For scaled analyses, we removed differences in size by choosing a reference model (A) and scaling the loads applied to the target model (B) to an identical force (*F*): surface area (SA) ratio using Eqn 1 (Dumont et al., 2009). For this, we used the surface area of the entire CT model (female SA: 322.934 mm^2^; male SA: 327.791 mm^2^). We selected the female specimen as the reference model (Fig. 2A, Table S1). It is important to note that the choice of reference model in FE comparative analysis is arbitrary. The resulting scaled force value applied to the male model was 203 mN.
(1)

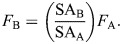


We used three metrics to compare the relative stabbing performance of green hermit bills: von Mises (VM) stress, total strain energy, and buckling load factor. VM stress is a good predictor of failure under ductile fracture, so, for a given load, structures with lower VM stress are less likely to fail, indicating that they are structurally stronger (Dumont et al., 2009). Total strain energy is a measure of the work expended in deformation, with lower values indicating stiffer structures that transmit force more efficiently (Dumont et al., 2009). In scaled analyses, to compare strain energy magnitudes, we adjusted them to the same force: volume (*V*) ratio using Eqn 2 (Dumont et al., 2009). Model volumes were: female (*V*_A_)=32.691 mm^3^ and male (*V*_B_)=33.042 mm^3^.
(2)

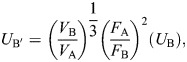
where *U*_B_ is the computed strain energy of the target model (male) and *U*_B′_ is the adjusted strain energy of the target model used for comparisons. This equation was only applied to the scaled models of the male individual.

Since long, slender structures subjected to axial loads may fail through buckling (i.e. sudden change in shape owing to loss of stability under compressive loads; Wainwright et al., 1976; Marcé-Nogué, 2022), we assessed buckling resistance using eigenvalue buckling analysis (Cook et al., 2002), wherein the minimum eigenvalue corresponds to the buckling load factor. Higher buckling load factor values indicate greater resistance to buckling failure. We note that these values should only be considered in a comparative context; they do not represent absolute measures of bill mechanical performance.

### Data analysis

To assess sexual dimorphism in bill shape of green hermits, we performed Procrustes ANOVA with permutation procedures (Goodall, 1991) using the ‘procD.lm’ function in Geomorph. With the same function, we explored the relationship between bill curvature and sharpness (predictor variables) to bill shape (response variable), as well as between bill length and surface area (predictor variables) to bill centroid size (response variable). We did not test for allometric effects, as all specimens were adults. We used non-parametric Mann–Whitney *U*-tests to evaluate intersexual differences in the bill-tip sharpness (ratio and included angle), bill curvature, arc length, and surface area. We performed Spearman correlations to test for the relation between our sharpness ratio and the included angle, and between both sharpness metrics and PC1 of the bill shape space. All calculations, as well as morphometric and statistical analyses, were performed in R version 4.4.1 (http://www.R-project.org/).

We evaluated relative bill stabbing performance by comparing the magnitudes of VM stress, total strain energy, and buckling load factor between the sexes. These single-value metrics are useful indicators of the biomechanical performance of a model. We summarized VM stress using the peak stress and the mesh-weighted arithmetic mean of the stress (MWAM). Artificially high stress values, known as numerical singularities, frequently occur in finite element models of complex shapes, potentially obscuring the identification of biologically relevant results (Marcé-Nogué, 2020). We excluded the top 2% stress values to avoid these artifacts, using the 98th percentile as the peak VM stress – an approach applied by previous studies (Dumont et al., 2014; Habegger et al., 2019; Sansalone et al., 2024). Instead of the mean stress, we used MWAM because it accounts for differences in element volumes within the meshes (Marcé–Nogué et al., 2016; Murphy et al., 2024; Rowe and Rayfield, 2022). The distribution of VM stress was visualized using a variation of the inferno color map (i.e. plasma), as it offers increased accessibility for people with color vision deficiency and reduced distortion of the data (Lautenschlager, 2021).

### Ethics

We recorded Movie 1 under the permits from the University of Washington's Institutional Animal Care and Use Committee (IACUC protocol number: 4498-05), and the Corporación Autónoma Regional de Cundinamarca (permit number: 50227001473).

## RESULTS AND DISCUSSION

### Bill sexual dimorphism

Our bill-tip sharpness metrics indicate that green hermit males possess dagger-like bill-tips, candidate traits to be considered intrasexually selected weapons (Rico-Guevara and Hurme, 2019), as they are present or more developed in one sex, but their use as contact-contest tools during same-sex agonistic encounters warrants further field-based investigation. However, given that lekking species of the genus *Phaethornis* exhibit similar aggressive behaviors (Snow, 1974; Stiles and Wolf, 1979), and the closely related long-billed hermit employs its bill as a weapon (Rico-Guevara and Araya-Salas, 2015), we expect that the green hermit also does so. Whether green hermit daggers enhance reproductive success through lekking territory tenure remains unexplored.

We found significant intersexual differences in green hermit bill shape and size. Sexual dimorphism aligned closely with PC1 of the bill shape space (Fig. 2A), showing a clear separation between sexes along PC1 (69.69%) and high overlap along PC2 (16.76% of total shape variation). Intersexual differences accounted for 45% of bill shape variation (*R*^2^=0.45, *P*=0.004), with bill curvature contributing substantially to this variation (*R*^2^=0.57, *P*=0.001), where male bills towards the negative side of PC1 were straighter and female bills towards the positive side were curvier (Fig. 2B). Males had significantly straighter (*W*=64, *P*<0.001) and longer bills than females (*W*=11, *P*=0.03; Fig. 2C), as previously seen in the green hermit and other hermits (Bleiweiss, 1999; Temeles et al., 2010; Berns and Adams, 2013). Males had significantly sharper bill-tips (sharpness ratio: *W*=8, *P*=0.01; included angle: *W*=1, *P*=0.0003; Fig. 2B,C; Fig. S1) and greater bill surface area (*W*=0, *P*=0.0012), with the sharpness ratio explaining 13% of bill shape variation based on a Procrustes ANOVA (although not significantly, *R*^2^=0.13, *P*=0.161). The sharpness ratio did not correlate significantly with the included angle (Spearman's *r*=−0.41, *P*=0.12), so future work should assess whether the sharpness ratio is a reliable predictor of puncture ability. Among the two measures of sharpness, PC1 of the bill shape space correlated more with the included angle (Spearman's r=0.72, *P*=0.003), than with the sharpness index (Spearman's *r*=−0.43, *P*=0.13). Bill centroid size had a stronger association with bill arc length (*R^2^*=0.94, *P*=0.001), than with surface area (*R*^2^=0.69, *P*=0.001).

Our findings align with research on hummingbird bill dimorphism previously invoking ecological causes (Bleiweiss, 1999; Temeles et al., 2010). In species-poor environments, the absence of competitors enables males and females to occupy separate ecological niches that would otherwise be filled by closely related species, with bills of distinct curvature allowing sexes to specialize on feeding from different flowers (Temeles et al., 2010). Longer bills enable one sex to access a greater variety of floral resources, while shorter, straighter bills enhance efficiency in feeding from small, localized patches (Bleiweiss, 1999). However, intrasexual selection could also drive bill morphology by enhancing combat effectiveness during territory defense. Longer and straighter bills may increase reach during confrontations, while sharper bill-tips increase damage (Rico-Guevara and Araya-Salas, 2015; Rico-Guevara et al., 2019). Furthermore, with all other dimensions remaining constant, an increase in bill surface area leads to a larger cross-sectional area, which in turn enhances bill bending strength (Wainwright et al., 1976).

### Bill stabbing performance

**Fig. 3.**
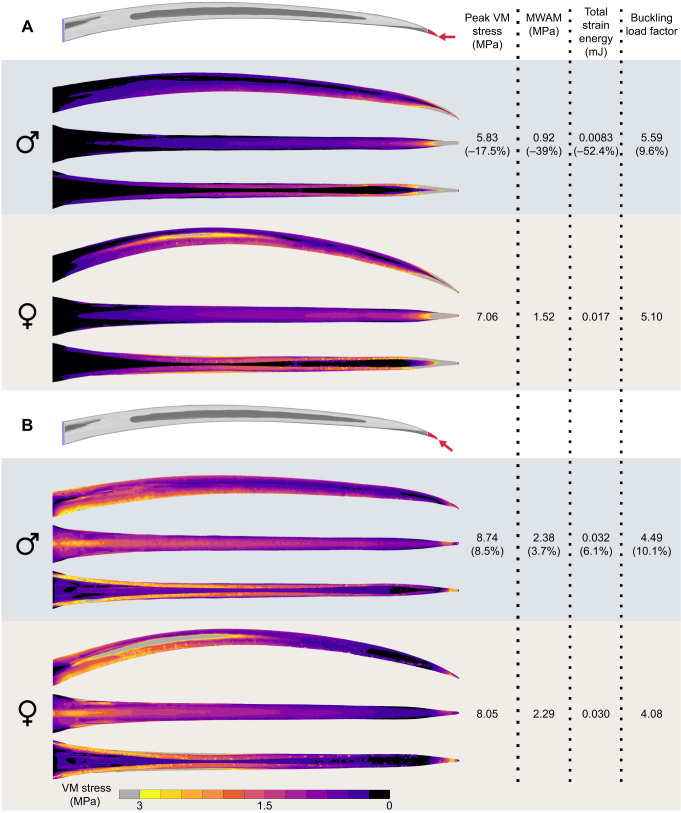
**Bills of male *Phaethornis guy* are stronger and stiffer under horizontal scaled loading relative to female bills.** Scaled finite element analysis (FEA) results of stabbing simulation at two load angles. (A) Horizontal and (B) parallel to the bill-tip axis. Left: von Mises stress distribution on male and female bills in lateral, dorsal and ventral views. Warmer colors (and gray at the extreme) indicate regions of higher stress. Right: Comparisons of biomechanical performance metrics, including peak and mesh-weighted arithmetic mean von Mises stress, total strain energy, and buckling load factor. The percentage difference of male values relative to the female is shown in parentheses.

We found that the straighter bills of male green hermits confer biomechanical advantages for horizontal stabbing. In the scaled analysis, the male bill expended 52.4% less energy in deformation, the largest difference observed across all biomechanical metrics (Fig. 3A), indicating that male bills are more efficient at transmitting stabbing forces at horizontal angles than female bills are. Furthermore, male bills showed lower breakage risk, with reductions of 39% in MWAM and 17.5% in peak von Mises stress relative to levels in female bills. Morphologically, these individuals differed most in included angle (69% lower in the male), which has been shown to be a good predictor of puncture performance (Crofts et al., 2019) and the sharpness ratio (16% higher in the male), whereas arc:chord ratio, arc length, outer surface area and centroid size differed by less than 3%. In contrast, when stabbing at the angle parallel to the bill-tip, performance was similar between the sexes (stress and strain energy differences <10%; Fig. 3B). Both sexes exhibited comparable buckling resistance under both loading angles (∼10% differences; Fig. 3). Relative to females, males did not experience a substantial loss of stabbing performance across tested angles. Instead, their performance increased under horizontal loading, suggesting that the straighter bills of males can accommodate a greater range of potential stabbing angles, requiring less precision during fighting.

Biomechanical performance (FEA results) is expected to vary on a gradient along PC1 of the morphospace, such that intermediate bill morphologies will show intermediate stabbing performance between the female and male extremes. We hypothesize that male positions along this axis could reflect their reproductive strategy at leks: territorial males would cluster toward the extreme associated with higher stabbing performance (straighter bills), whereas non-territorial ‘floaters’ would lie closer to the center, nearer to female morphologies (curvier bills). Male long-billed hermits display two distinct reproductive strategies at leks which are related to differences in bill morphology (Rico-Guevara and Araya-Salas, 2015): on one hand, there are territorial males, which defend a set of perches within the lek and have pointier and more elongated daggers, and, on the other hand, there are non-territorial ‘floaters’ that do not defend territories, instead they stay in the lek periphery and potentially follow females attracted to the lek, they in turn have less pointy and shorter daggers.

In the unscaled analyses, male bills showed greater biomechanical performance in both stabbing scenarios (in all metrics; Fig. S2), with larger intersexual differences in strength and stiffness during horizontal loading. Lower forces were applied to the male bill in the unscaled compared with the scaled analyses, reflecting the fact that sharper tools can penetrate target materials with lower forces owing to the reduced area over which the force is concentrated (Anderson, 2018). Thanks to their sharper bill-tips, male green hermits may minimize the forces required to puncture (as observed in long-billed hermits; Rico-Guevara and Araya-Salas, 2015), subjecting their bills to lower biomechanical demands. Direct measurements of stabbing forces during lekking interactions would provide valuable insights into the actual demands experienced by bills during combat.

Across models, parallel loading consistently produced higher stress and strain energy, and lower buckling resistance than horizontal loading. During horizontal loading, bills experienced downward bending, with regions of high stress concentrated at the bill-tip and distributed along the tomium and culmen, dissipating longitudinally toward the maxillary base. In parallel loading, bills exhibited upward bending, with high stress concentrated at the base and dissipating toward the tip, with a drop in stress around the distal quarter of the bill and a localized increase at the loading point. These differences in stress propagation indicate that bills experience distinct mechanical challenges depending on the stabbing angle, potentially influencing fighting strategies. An important consideration is that hummingbirds exhibit cranial kinesis, with hermits being the only group so far documented to show prokinesis, slight rotation of the bill at the craniofacial hinge (Movie 1; Zusi, 2013 observed in *P. guy* during manipulation of fresh specimens). The bones and articulations allowing cranial kinesis could interact with the elevated stresses observed at the bill base under parallel loading, for example, by absorbing or redirecting loads into surrounding cranial elements, decreasing the probability of tissue damage. Stress dissipation into the posterior cranium has been documented in woodpeckers (Jung et al., 2019), where impact forces at the bill-tip propagate through the jugal bones toward the spine, while stress near the brain remains low.

Green hermits may prefer to stab opponents at angles horizontal to the bill axis for different reasons. First, as shown here, bills transmit forces more efficiently, experience less stress and are more resistant to buckling during horizontal stabbing (Fig. 3; Fig. S2). In contrast, when loads are applied in parallel to the bill-tip, bills exhibit lower strength and stiffness. This pattern aligns with findings for other curved piercing tools. Scorpion stingers and spider chelicerae have optimal piercing angles that minimize structural stress and strain energy (van der Meijden and Kleinteich, 2017; Bar-On et al., 2014). Notably, these angles consistently differ from the angle parallel to their tips. Second, horizontal stabbing is a less complex behavior. It occurs when the attacker flies toward the opponent, and its bill-tip meets the target's body. Meanwhile, parallel stabbing requires more intricate motions, wherein the attacker must rotate its head ventrally following the curvature of the bill while making contact. It is noteworthy that the angle of attack during puncturing has little influence on the damage inflicted on the target (Zhang et al., 2024). Thus, hummingbirds may adjust their attack angles to minimize the stress and deformation experienced by their bills without compromising the inflicted damage. While our results suggest a biomechanical advantage for horizontal stabbing, whether male green hermits prefer this angle during combat requires confirmation through behavioral studies.

### Concluding remarks

Our results suggest that male green hermits possess weaponized bills with sharp, dagger-like tips, a putatively intrasexually selected trait previously identified only in long-billed hermits and one of the few known examples of avian male weaponry (Rico-Guevara and Araya-Salas, 2015; Rico-Guevara and Hurme, 2019). Furthermore, we show that the straighter shape of male bills efficiently transmits stabbing forces at a horizontal angle of attack (parallel to the bill axis), supporting the potential role of intrasexual selection in the evolution of bill dimorphism.

Although 3D models have been widely used to study bill shape across bird taxa (e.g. Cooney et al., 2017), they had not, to our knowledge, been used to examine bill sexual dimorphism. Likewise, finite element analysis has been used in birds to evaluate biomechanical performance in food processing and cavity excavation (Attard et al., 2016; Chhaya et al., 2023), and in other taxa, such as arthropods and mammals, to study weapon function (McCullough et al., 2014; Klinkhamer et al., 2019), but this study represents the first application of FEA to address questions related to sexual selection in birds. These methods could serve as a framework for studying the presence and function of bill weaponry across the avian class.

We used homogeneous material properties to isolate the effects of shape on biomechanical performance. However, bill elasticity may vary along its length, as suggested by flexural rigidity estimates in hummingbirds (Rico-Guevara et al., 2024). Additionally, male long-billed hermit bill-tips feel stiffer to the touch (Rico-Guevara and Araya-Salas, 2015), possibly due to differences in mineralization. Stiffer bill-tips could enhance resistance to stabbing-induced stress but may reduce feeding efficiency, as maxillary-tip bending plays a key role in nectar extraction (Rico-Guevara et al., 2023, 2024). Studies on hummingbird bill material properties and the potential trade-off between stabbing performance and feeding efficiency are warranted.

Future work should explore bill-tip sharpening across hummingbirds, examining its relation to lekking and territoriality (Stiles and Wolf, 1979; Sargent et al., 2021), to better understand the distribution and evolutionary drivers of this trait. Of particular interest is the case of female hummingbirds in species with feeding territoriality, as their parental care duties may conflict with developing bills for stabbing. Sharp bill-tips could pose a risk of injury to nestlings during feeding (which involves deep insertion of the female's bill-tips into the chicks' throats), potentially limiting the evolution of this trait in females. However, sharp bill-tips could also provide an advantage in nest defense, helping females deter predators. If sharp bills do occur in female hummingbirds, it would be valuable to explore whether there are behavioral or morphological adaptations in females or nestlings to prevent injury. Detailed descriptions and quantification of hummingbird fighting behaviors are needed. Weapons are considered rare in birds, as the high energetic cost of flight may prevent their evolution or promote their loss (Menezes and Palaoro, 2022). However, the presence of weaponry in hummingbirds suggests that such traits can evolve as fine modifications of preexisting structures without increasing flight costs. Similar adaptations may have evolved in other avian lineages that exhibit combat behaviors, potentially making weapons more widespread in birds than previously recognized.

## Supplementary Material

10.1242/jexbio.250769_sup1 Supplementary information
