# Supplementary material for: Sharper, straighter, stiffer, stronger: sexually dimorphic bill shape enhances male stabbing performance in the green hermit hummingbird (Phaethornis guy)
Source: J Exp Biol. 2025 Nov 10;228(21):jeb250769. doi: 10.1242/jeb.250769 (PMC12669840; doi:10.1242/jeb.250769)
Supplement: Supplementary information [file jexbio-228-250769-s1.pdf]

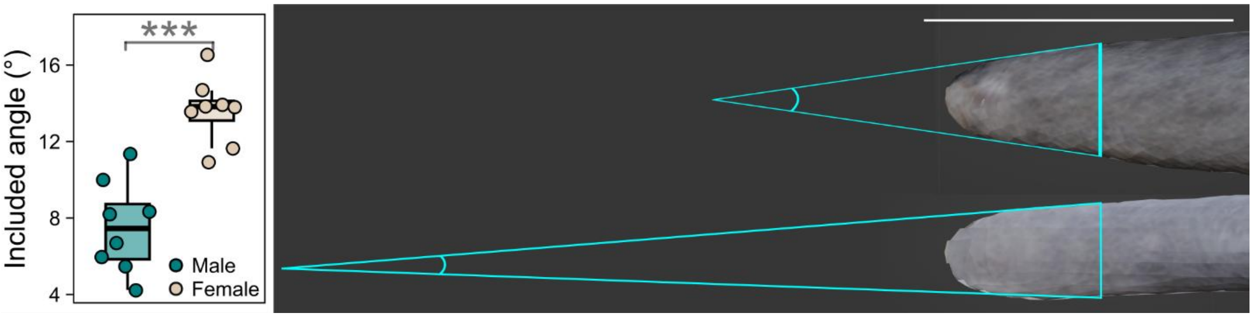

Fig. S1. Bill-tip sharpness measured as the tip included angle. Left: Boxplot showing intersexual comparison of included angle. Level of significance (Mann-Whitney U): ****P*<0.001. Right: Close-up of the bill-tip from the dorsal view showing the included angle measurement on female (top) and male (bottom) specimens. Bar: 1 mm.

Journal of Experimental Biology • Supplementary information

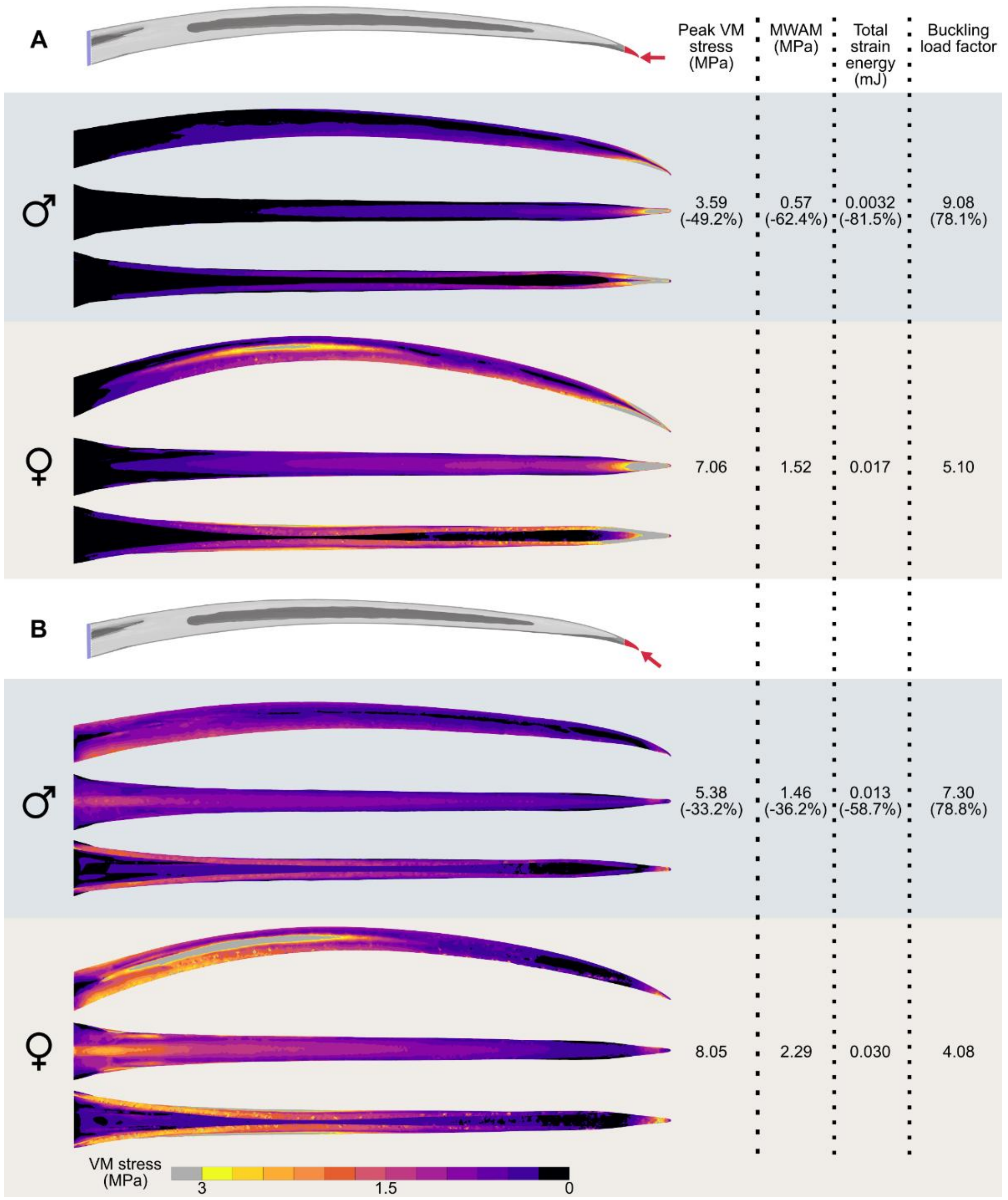

Fig. S2. Relative to females, bills of male *Phaethornis guy* were stronger, stiffer and more resistant to buckling under horizontal and parallel *unscaled* loading. Unscaled FEA results of stabbing simulation at two load angles: (A) horizontal and (B) parallel to the bill-tip axis. Left: Contour plots showing von Mises stress distribution on male and female bills in lateral, dorsal and ventral views. Warmer colors indicate regions of higher stress. Right: Comparisons of biomechanical performance metrics including peak and mesh-weighted arithmetic mean von Mises stress, total strain energy, and buckling load factor. Values in parentheses represent the percentage difference of male values relative to the female. Under both loading scenarios, male bills showed greater resistance to breaking and to buckling failure, and expended less energy in deformation.

Journal of Experimental Biology • Supplementary information

Table S1. Bill morphometric variables from photogrammetry models.

Specimen ID	Sex	Sharpness ratio	Included angle (°)	Arc: Chord	Arc length (mm)	Outer SA (mm²)	CS (mm)	PC1	PC2
UWBM-76887	M	0.53	8.3	1.019	40.8	172.85	160.2	-0.0103	-0.0035
**UWBM-76890**	M	0.54	6.6	1.017	41.2	174.08	161.8	-0.0228	-0.0093
UWBM-76893	F	0.38	11.6	1.027	40.1	168.37	158.0	0.0113	-0.0011
UWBM-76984	M	0.46	10.0	1.014	43.1	173.82	166.6	-0.0151	-0.0002
UWBM-108340	M	0.51	6.7	1.017	42.4	176.76	166.8	-0.0068	-0.0037
UWBM-108783	F	0.38	13.9	1.021	41.3	171.05	162.9	-0.005	-0.0031
UWBM-111316	F	0.49	14.7	1.029	39.6	160.35	155.1	0.0048	0.008
UWBM-112274	M	0.52	11.4	1.019	42.1	179.17	165.9	-0.0036	0.0071
UWBM-112313	F	0.44	16.6	1.025	39.5	170.59	155.9	0.0105	0.0049
UWBM-123358	M	0.48	5.4	1.019	43.1	-	-	-	-
UWBM-123451	M	0.46	6.0	1.019	40.8	175.07	162.0	-0.0116	0.0064
UWBM-123474	F	0.50	13.8	1.025	39.6	157.34	155.1	0.0106	-0.0065
UWBM-123475	F	0.43	10.9	1.030	40.8	-	-	-	-
UWBM-123546	F	0.44	13.6	1.033	41.6	-	163.4	0.0136	0.0056
**UWBM-123686***	F	0.47	16.5	1.031	41.0	170.38	162.3	0.0248	-0.0111
UWBM-125003	M	0.50	8.2	1.021	41.3	175.48	163.7	-0.0005	0.0063

Selected specimens for FEA are in bold. The asterisk marks the reference specimen for scaled FEA. M, male; F, Female; SA, outer maxillary surface area; CS, centroid size; PC, principal component of shape variation.

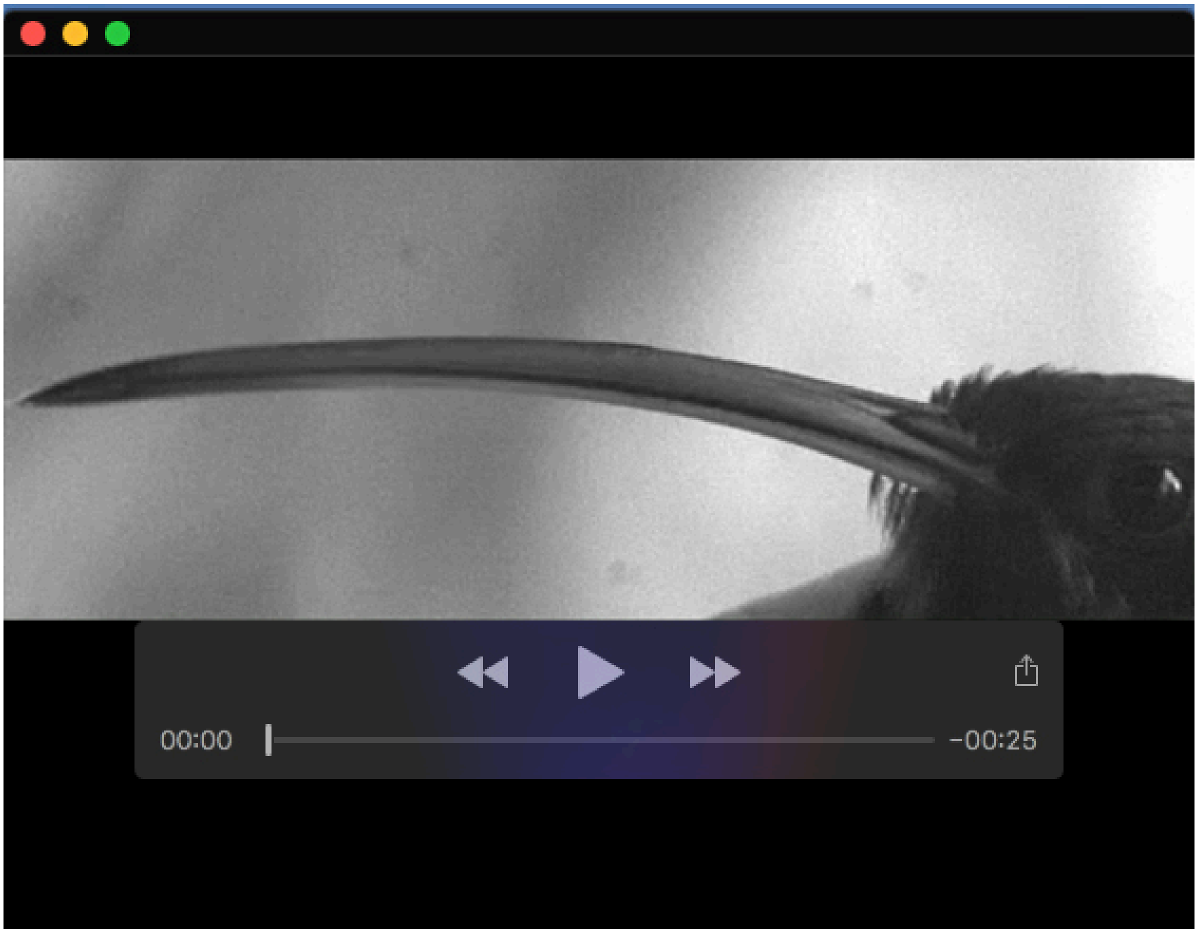

Movie 1. *Phaethornis guy* showing cranial kinesis.
